# The Effect of Short Term Vitamin D Supplementation on the Inflammatory and Oxidative Mediators of Arterial Stiffness

**DOI:** 10.4236/health.2014.612185

**Published:** 2014-06

**Authors:** David Martins, Yuan-Xiang Meng, Naureen Tareen, Jorge Artaza, Jae Eun Lee, Caroline Farodolu, Gary Gibbons, Keith Norris

**Affiliations:** 1 Charles R. Drew University, Los Angeles, USA; 2 Morehouse School of Medicine, Atlanta, USA; 3 Jackson State University, Jackson, USA; 4 National Heart, Lung, and Blood Institute, Bethesda, USA; 5 University of California Los Angeles, Los Angeles, USA

**Keywords:** Vitamin D, Overweight, Hypovitaminosis D

## Abstract

**Background:**

Vitamin D deficiency has been implicated as a potential risk factor for cardiovascular disease. The high rate of vitamin D deficiency (<30 ng/ml) exhibited by African Americans may account for some of the excess prevalence of cardiovascular morbidity and mortality in this vulnerable US population. Vitamin D supplementation may reduce the risk of cardiovascular disease by ameliorating the onset and progression of arterial stiffness, a strong predictor of cardiovascular mortality, usually assessed by pulse wave velocity and augmentation index. Very few prospective studies have evaluated the effect of vitamin D supplementation on the inflammatory and oxidative stress mediators of arterial stiffness.

**Method:**

In a double blind randomized placebo controlled study we evaluated the effect of a monthly dose of 100,000IU of vitamin D3 for three months on the level of serum 25(OH)D, intact parathyroid hormone (PTH), urinary isoprostane, adipocyte cytokine expression and arterial stiffness among 130 overweight and obese (BMI > 25) African Americans with elevated blood pressure (130 - 150/85 - 100 mmHg) and low serum vitamin D level (10 - 25 ng/ml).

**Results:**

There was a significant increase in the serum 25(OH)D levels to a mean level of 34.5 ng/ml (SD = 7.1) with the intervention (p < 0.001). The increase in 25(OH)D levels was associated with a significant decrease in the serum level of intact PTH (p = 0.02), mean urinary isoprostane (p = 0.02) and adipocyte cytokine expression. Although the increase in the 25(OH)D levels was not associated with any significant change in the Pulse Wave Velocity (PWV) in the overall study sample, it was associated with a significant decrease in the augmentation index among the participants with the highest tertile of urinary isoprostane (p = 0.007).

**Conclusion:**

We concluded that vitamin D supplementation increased serum 25(OH)D levels, decreased intact PTH level and the levels of select inflammatory and oxidative stress mediators of arterial stiffness. Longer term prospective studies are warranted to evaluate the effect of high dose vitamin D supplementation on arterial stiffness.

## 1. Background

The in-depth understanding of the effect of vitamin D on cardiovascular (CV) disease morbidity and mortality has been significantly impaired by the limited number of prospective studies, the suboptimal dosing of vitamin D across most of the available prospective studies, and a limited understanding of the CV mechanism through which vitamin D may mediate health benefit [1]. Even though the serum level of 25 hydroxy-vitamin D (25OHD) has been used to assess vitamin D intake, and vitamin D sufficiency has been defined as 25OHD serum levels of 30 - 100 ng/ml [2], very few studies measured the serum level of 25OHD and the dosages of vitamin D employed in most of the studies were below the recommended 1500 - 2000IU required to keep the serum level of 25OHD consistently above 30 ng/ml.

Vitamin D insufficiency defined as 25OHD serum levels of 21 - 29 ng/ml is nearly twice as common among African Americans and significantly associated with increased rates of both CV risk factors, and CV disease [3]. Hyperpigmentation of the skin and excess prevalence of overweight and obesity could among other things pre-dispose African Americans to vitamin D insufficiency and the increased rates of CV risk factors and CV disease associated with vitamin D insufficiency may be related in part to the excess prevalence of overweight and obesity associated with vitamin D insufficiency. The extent to which the serum 25(OH)D levels of 30 ng/ml or more recommended for optimum bone health and extra-skeletal benefits of vitamin D can attenuate the disproportionate burden of CV risk factors and CV disease associated with hyperpigmentation and excess prevalence of overweight and obesity is unknown.

Vitamin D supplementation may reduce the risk of cardiovascular morbidity and mortality by ameliorating the onset and progression of arterial stiffness, a strong predictor of cardiovascular mortality [4] usually assessed by pulse wave velocity and augmentation index. Vitamin D supplementation for four months with 2000IU/d was shown to raise the serum level of 25OHD above 30 ng/ml and to significantly reduce arterial stiffness among African-American teenage boys and girls [5]. Adult African Americans have been shown to exhibit the lowest levels of serum 25(OH)D in the US [6] but the effect of adequate vitamin D supplementation on arterial stiffness and subsequent cardiovascular risk among adult African American men and women is not well established. The purpose of this study was to evaluate the effect of a short term high dose vitamin D supplementation on the inflammatory and oxidative stress mediators of arterial stiffness as determined by pulse wave velocity and augmentation index.

## 2. Methods

### 2.1. Study Population

The participants for this study were recruited primarily from affiliated medical clinics at Charles R. Drew University and Morehouse School of Medicine. A total of 130 (65 participant per site) male and female hypertensive African Americans, aged 18 - 70 years with blood pressure less than 160/100 mmHg and serum levels of 25(OH)D between 10 ng/ml and 25ng/ml were included in the study. The exclusion criteria included poorly controlled high blood pressure (SBP > 160 or DBP > 100), diabetes (FBS > 125 mg/dl or HbA1c > 6.5%), severe vitamin D deficiency {25(OH)D < 10 ng/ml}, chronic kidney disease (eGFR < 45 ml/min), hypercalcemia (serum calcium > 10.5 mg/dl), abnormal liver function tests, recent (<6 months) hospitalization for myocardial infarction, stroke or congestive heart failure, history of kidney stones, allergy to oral vitamin D or microcrystalline cellulose, immunosuppressive therapy, chronic steroid therapy and treatment with non-steroidal anti-inflammatory drugs. Demographic information and a brief clinical history was obtained by the site investigators. Race/ethnic identification was reported by the participants. The study was approved by each of the local institutional review boards, all participants signed a written informed consent document.

### 2.2. Study Design

This study was a randomized double-blinded placebo-controlled clinical trial with a screening period; a twelve-week intervention phase and an end of study follow up. Participants were evaluated for eligibility for participation in the study during the screening period. A brief medical history and a limited physical examination that included the measurement of height (cm), weight (kg) and waist circumference and the calculation of the body mass index was performed and blood was drawn for serum 25(OH)D level, pregnancy test, intact parathyroid (iPTH) level (pmol/L), a complete blood count and a chemistry panel that included liver and renal functions and levels of glucose, hemoglobin A1c, calcium, phosphorus and albumin. Eligible participants were randomized to receive a monthly dose of 100,000IU of vitamin D_3_ or placebo for three months in the twelve-week intervention phase. Participants were followed up on site and blood was drawn for serum 25(OH)D, iPTH and calcium levels at 4, 8 and 12 weeks. At the end of the study, participants returned for a final brief history and a limited physical examination and those randomized to placebo were given a 3-month supply of oral vitamin D with a recommendation to follow up with their primary care providers.

### 2.3. Study Procedures

#### 2.3.1. Vascular Function Studies

Vascular and endothelial functions were assessed at baseline, week 8 and week 12 visits. Vascular function was assessed by radial artery tonometry and calculation of the augmentation index using the SphygmoCor family of products that allows a non-invasive measurement of the pressure wave in the ascending aorta from an external measurement taken at the radial artery. The endothelial function was assessed by percent flow mediated dilation using the Endopat non-invasive vascular finger plethysmography.

#### 2.3.2. Fat Biopsy

Pre- and post-intervention biopsies of subcutaneous fat were performed in a subset of the study participants (n = 10) to evaluate the effect of vitamin D supplementation adipocyte expression of select cytokines such as CD40 ligand, a transmembrane protein structurally related to Tumor Necrosis Factor alpha and implicated in platelet activation and thrombus formation [7], Growth Regulated Oncogene (GRO) Alpha, an Interleukin 8-related cytokine associated with chemotactic properties for human neutrophil and basophil leukocytes [8], and soluble Intercellular Adhesion Molecule-1 (sICAM-1), a biomarker of inflammatory process associated with endothelial damage and platelet activation. A separate informed consent was obtained for subcutaneous fat biopsy and participants with history of keloid formation and bleeding diathesis were excluded.

### 2.4. Statistical Analysis

All analyses were performed using SAS version 9.2 (SAS Institute Inc.). Baseline characteristics were compared between the two groups using Chi-Square tests for categorical variables and t-tests for continuous variables. We employed the maximum-likelihood mixed-effects repeated-measures model in the analysis of efficacy of the intervention. The effect of the vitamin D supplementation on serum 25(OH)D was determined by the interaction term between the change of the endpoints and study arms in order to test if there is any difference in change of the outcome between study groups. Nonlinear effects of the experiment on response variables over time were investigated using cubic mixed models. All statistical models included site ID as a covariate to control for the potential for differential effects across sites. The analyses were conducted on the intention to treat population and included all randomized participants. Although the mixed model without imputation was more powerful than other imputation methods, the multiple imputation method is also advantageous for dealing with missing values in longitudinal studies [9]. We therefore compared the results from two methods but no notable difference was found.

## 3. Results

**Figure 1:**
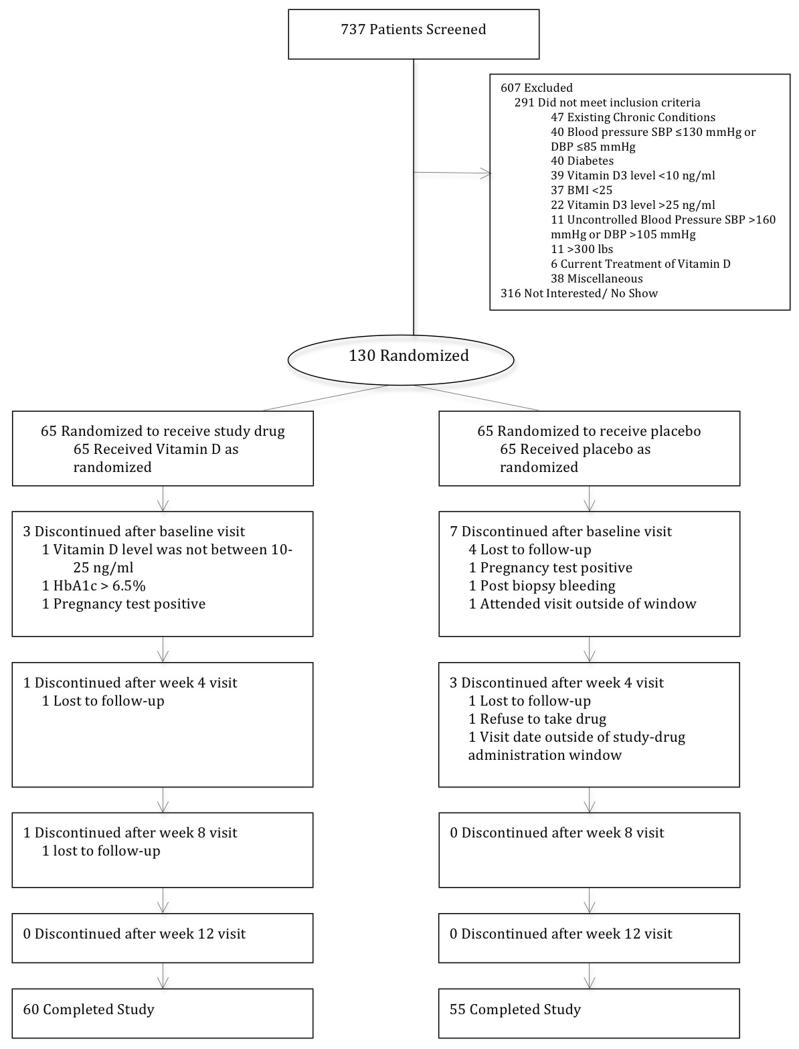
Study consortium diagram.

**Figure 2:**
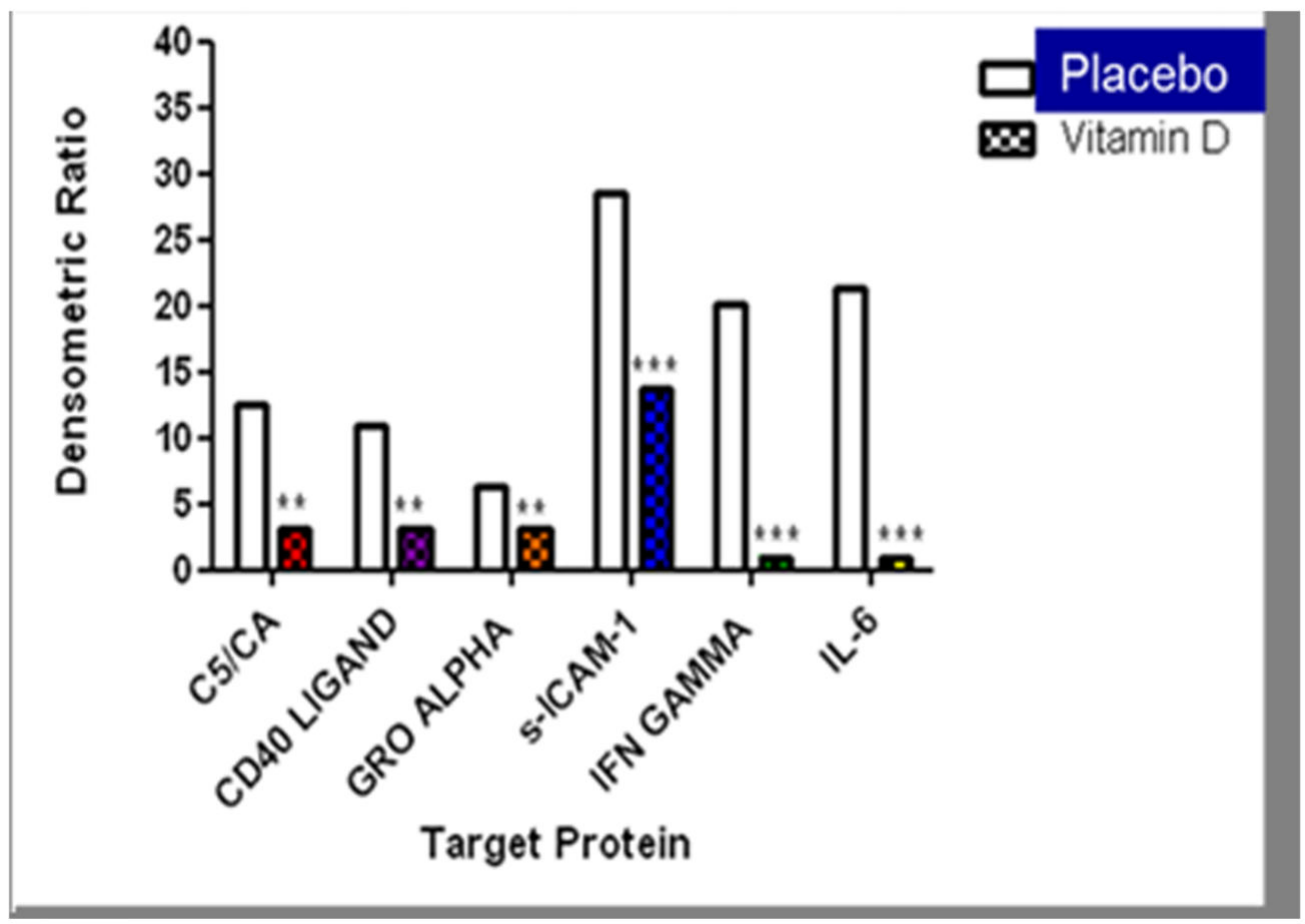
Adipocyte cytokine expression profile: Placebo versus vitamin D.

**Figure 3:**
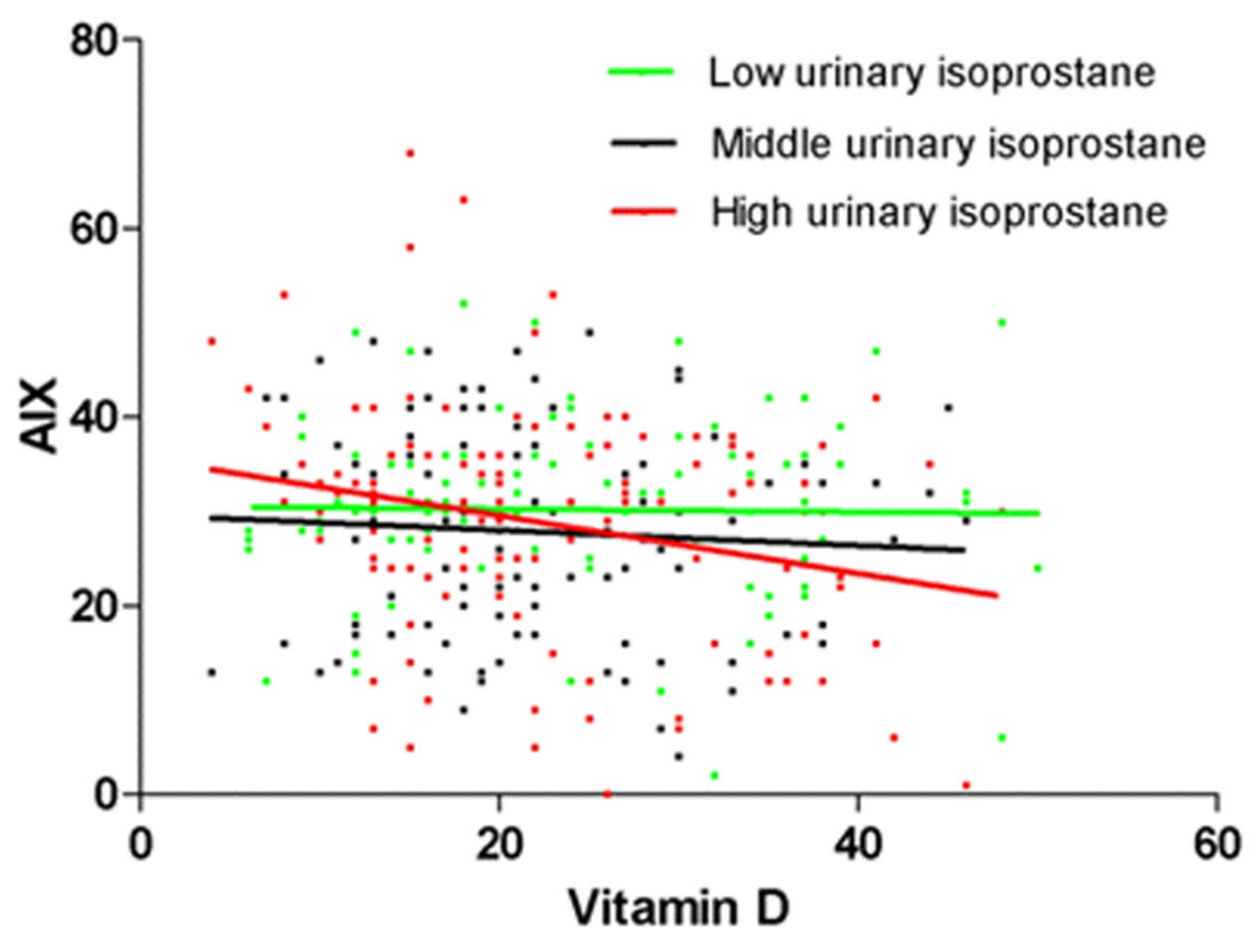
Augmentation index and vitamin D supplementation by tertiles of urinary isoprostane. p = 0.007 for the highest tertile.

A total of 737 participants from the two study sites were screened between September 2009 and August 2011. We excluded 607 participants that did not meet the inclusion criteria and of the 130 participants that were enrolled and randomized to receive 100,000IU of Vitamin D or placebo, 115 completed the study as depicted in Figure 1. There were no significant differences in the demographics and baseline characteristics of the participants between groups. Sixty percent of the participants were male, 70% were between 40 and 59 years and 78% were obese with high waist circumference measurements (Table 1). The monthly vitamin D dose of 100,000IU was associated with a rise in the serum 25(OH)D levels to a mean level of 34.5 ng/ml (SD = 7.1) in 12 weeks. While the serum levels of 25(OH)D were higher in the summer months than the rest of the year in both the treatment {17.9 (4.7) versus 15.9 (5.1), p = 0.16} and placebo {18.2 (4.0) versus 16.3 (5.7) p = 0.12} groups the differences were not statistically significant. The increase in the serum 25(OH)D levels was associated with a decrease in the serum level of intact PTH and urinary isoprostane (Table 2) as well as a decrease in the adipocyte cytokine expression (Figure 2). Although there was no change in the Pulse Wave Velocity (PWV) with the increase in the 25OHD levels in the overall study sample at 3 months, PWV decreased significantly (p = 0.007) with the increase in the serum 25OHD levels among the study participants with the highest level of urinary isoprostane (Figure 3).

## 4. Discussion

Vitamin D insufficiency defined as 25OHD serum levels of 21 - 29 ng/ml affects nearly 50% of the nation and over 80% of African Americans [10] and is significantly associated with increased rates of both CV risk factors, and CV disease [11]. However, the results of vitamin D supplementation studies for cardiovascular benefit have been conflicting partly because of the limited dose and duration of vitamin D supplementation in most of the studies along with the poor understanding of the mechanism by which vitamin D may mitigate CV disease and risk factors.

Vitamin D is modified by 25-hydroxylase in the liver to form 25OHD and the nutritional status of vitamin D has always been assessed by the circulating level of 25OHD [12]. The Clinical Guidelines for the Evaluation, Treatment and Prevention of Vitamin D deficiency by the Endocrine Society defined vitamin D sufficiency as serum 25OHD levels of 30 - 100 ng/ml. At the mean serum 25OHD level of 34.5 ng/ml (*SD* = 7.1) in this study we demonstrated a significant reduction in the expression and activation of the inflammatory and oxidative stress mediators of arterial stiffness.

The decrease in the PWV with the increase in the serum 25OHD among the participants with the highest tertile of urinary isoprostane suggests that the cardiovascular benefit of vitamin D supplementation may be more apparent in high risk populations with excess burden of oxidative stress. The absence of a decrease in the PWV with the increase in the serum 25OHD in the overall study sample may indicate that vitamin D supplementation in the short term has no cardiovascular benefit or that the levels of serum 25OHD achieved in this study are too low for the cardiovascular benefit of vitamin D supplementation.

The adequacy of vitamin D has hitherto been benchmarked to bone mineralization and serum 25OHD levels above 20 ng/ml (50 nmol/l) have been recommended for the prevention of osteoporosis [13]. However, it is becoming increasingly apparent that serum 25OHD levels above 30 ng/ml (75 nmol/l) may be required for optimum bone health [14] and the extra-skeletal benefits of vitamin D [15]. The steep rise in the serum levels of parathyroid hormone (PTH) when the serum levels of 25OHD fall below 30 ng/ml lends additional support to the physiologic insufficiency of serum levels of 25OHD below 30 ng/ml [16].

The cardiovascular effects of vitamin D may be mediated through the PTH axis and linked to reduced concentrations of serum ionized calcium, especially in low-renin human hypertension prevalent among African Americans [17]. Both PTH and its related protein (PTHrP) receptor have been reported in vascular smooth-muscle cells [18]. Coronary microvascular dysfunction [19] and impaired flow-mediated vasodilation of the brachial artery [20] induced by primary hyperparathyroidism have been shown to improve significantly after parathyroidectomy. Although there was no change in the finger plethysmography with the rise in serum 25OHD levels in this study, the decrease in the serum level of iPTH correlated significantly with a decrease in the PWV (results not shown) in the overall study sample (p = xx).

The active metabolite of vitamin D, 1,25-dihydroxyvitamin D (1,25OH2D), usually formed from 25OHD by 25-hydroxyvitamin D 1α-hydroxylase in the kidney [21] has also been shown to act on the vitamin D nuclear receptor (VDR) and activate the recruitment of cofactors into an ill-defined complex that binds to vitamin D response elements in the promoter region of target genes to regulate gene transcription [22]. The molecular events that occur in target tissues when stimulated by 1,25OH2D are complex and dependent on numerous and poorly understood membrane proteins, cytosolic factors, transcription co-activators and co-repressors [23]. In a gene chip microarray analysis of the modulatory effects of vitamin D analogs on the human coronary artery smooth muscle cells, a total of 176 target genes were identified of which 115 were up-regulated and 61 down-regulated [24]. In this study we extracted protein from human adipocytes and use the proteome profiler arrays to demonstrate changes in select cytokine expression profiles with vitamin D supplementation.

The presence of a high-affinity vitamin D receptor (VDR) in many tissues suggests that vitamin D controls a wide range of physiological functions including regulation of the synthesis and secretion of parathyroid hormone (PTH) and modulation of the immune response [25]. The immunomodulating effects of 1,25OH2D have been shown to occur via the VDR-signaling pathway that involve T cells, B cells, dendritic cells and macrophages [26] and may play a role in the atherosclerotic process by mitigating the accumulation of cells and extracellular matrix between the endothelium and the smooth muscle cell wall. In addition, the endothelin receptor type B (ETB) responsible for endothelial cell survival, the release of nitric oxide and prostacyclin as well as the inhibition and clearance of endothelin-1 (ET-1) has been shown to be up-regulated by the vitamin D analogs [27].

The ET-1 is usually induced in the endothelial and vascular smooth muscle cells in response to oxidized LDL cholesterol and has been associated with smooth muscle cell contraction, inflammatory cell proliferation and migration via the activation of endothelin receptor type A. The up-regulation and activation of ETB by vitamin D is apt to mitigate the ET-1 mediated vascular response to oxidative injury [28] and may account in part for the improved vascular function with the increase in serum 25OHD levels among the participants with the highest tertile of urinary isoprostane. Although vitamin D has been postulated to mediate its cardiovascular effect by modulating the mediators of inflammation and oxidative stress, this is the first time the vascular response to vitamin D supplementation is directly linked to a marker of oxidative stress in humans. The findings from this study suggest that the cardiovascular benefit of vitamin D may require near maximum serum 25OHD levels of vitamin D sufficiency and may be more apparent in a subset of the population with an excess burden of oxidative stress.

## Figures and Tables

**Table 1:** Baseline Characteristics by Treatment Groups

	All	Placebo	Vitamin D	p value
Age, n (%)				
18-39y	20(15.4)	10(15.4)	10(15.4)	0.8756
40-59y	92(70.8)	47(72.3)	45(69.2)	
60y and above	18(13.9)	8(12.3)	10(15.4)	
Gender, n (%)				
Male	79(60.8)	38(58.5)	41(63.1)	0.5900
Female	51(39.2)	27(41.5)	24(36.9)	
Body Mass Index Categories, n (%)				
25.0-29.9	30(23.1)	18(27.7)	12(18.5)	0.2117
> 30	100(76.9)	47(72.3)	53(81.5)	
Waist Circumference Groups, n (%)				
Low (<102 for male and <88 for female)	29(22.3)	17(26.2)	12(18.5)	0.2922
High (>=102 for male and >=88 for female)	101(77.7)	48(73.9)	53(81.5)	
Blood Pressure (systolic), mean (SD)	126.8(15.7)	128.5(15.2)	125.2(16.1)	0.2215
Blood Pressure (Diastolic), mean (SD)	82.7(11.1)	84.5(10.5)	80.9(11.4)	0.0667
24-hour Blood Pressure (Systolic), mean (SD)	130.1(15.4)	131.4(15.4)	128.8(15.5)	0.3418
24-hour Blood Pressure (Diastolic), mean (SD)	84.5(10.8)	85.6(11.0)	83.4(10.5)	0.2584
Augmentation Index, mean (SD)	29.6(11.6)	31.0(12.0)	28.2(11.2)	0.1824
Urine Isoprostane, mean (SD)	14.6(13.8)	14.9(15.8)	14.4(11.6)	0.8251
Baseline Laboratory, mean (SD)				
Serum 25(OH)D level, nmol/L	16.8(5.1)	16.5(5.0)	17.0(5.2)	0.4488
Serum Calcium level, mg/dl	9.4(0.4)	9.3(0.4)	9.4(0.3)	0.2455
Intact PTH level, pmol/L	46.7(27.7)	49.9(33.6)	43.4(19.9)	0.1817

**Table 2:** Clinical and Laboratory Response to Vitamin D by Treatment Groups

Characteristics	Placebo			Vitamin D treated		
	Baseline	12 Weeks	p value	Baseline	12 Weeks	p value

Blood Pressure (systolic), mean (SD)	128.5(15.2)	125.8(13.3)	0.1815	125.2(16.1)	126.9(15.0)	0.5939
Blood Pressure (Diastolic), mean (SD)	84.5(10.5)	82.2(9.2)	0.1794	80.9(11.4)	81.1(12.0)	0.8898
24-hour Blood Pressure (Systolic), mean (SD)	131.4(15.4)	128.4(14.0)	0.2474	128.8(15.5)	127.4(16.4)	0.4155
24-hour Blood Pressure (Diastolic), mean (SD)	85.6(11.0)	83.9(10.1)	0.649	83.4(10.5)	82.0(11.6)	0.3602
Augmentation Index, mean (SD)	31.0(12.0)	29.3(11.0)	0.2834	28.2(11.2)	27.6(11.0)	0.585
Urine Isoprostane, mean (SD)	14.9(15.8)	11.7(12.1)	0.6806	14.4(11.6)	11.0(9.4)	0.0173
Laboratory, mean (SD)						
Serum 25(OH)D level, nmol/L	16.5(5.0)	17.2(6.4)	0.5319	17.0(5.2)	34.5(7.1)	<.0001
Serum Calcium level, mg/dl	9.3(0.4)	10.0(7.5)	0.6364	9.4(0.3)	9.4(0.4)	0.5234
Intact PTH level, pmol/L	49.9(33.6)	49.7(37.6)	0.3125	43.4(19.9)	37.5(16.2)	0.0063

## References

[1] Elamin MB, Abu Elnour NO, Elamin KB, Fatourechi MM, Alkatib AA, Almandoz JP, Liu H, Lane MA, Mullan RJ, Hazem A, Erwin PJ, Hensrud DD, Murad MH, Montori VM (2011). Vitamin D and Cardiovascular Outcomes: A Systematic Review and Meta-Analysis. The Journal of Clinical Endocrinology & Metabolism.

[2] Holick MF, Binkley NC, Bischoff-Ferrari HA, Gordon CM, Hanley DA, Heaney RP (2011). Evaluation, Treatment, and Prevention of Vitamin D Deficiency: An Endocrine Society Clinical Practice Guideline. The Journal of Clinical Endocrinology & Metabolism.

[3] Zadshir A, Tareen N, Pan D, Norris KC, Martins D (2005). The Prevalence of Hypovitaminosis D among US Adults: Data from the NHANES III. Ethnicity & Disease.

[4] Hansen TW, Staessen JA, Torp-Pedersen C (2006). Ambulatory Arterial Stiffness Index Predicts Stroke in a General Population. Journal of Hypertension.

[5] Dong Y, Pollock N, Stallmann-Jorgensen IS, Gutin B, Lan L, Chen TC (2010). Low 25-Hydroxyvitamin D Levels in Adolescents: Race, Season, Adiposity, Physical Activity, and Fitness. Pediatrics.

[6] Zadshir A, Tareen N, Pan D, Norris K, Martins D (2005). The Prevalence of Hypovitaminosis D among US Adults: Data from the NHANES III. Ethnicity & Disease.

[7] Henn V (1998). CD40 Ligand on Activated Platelets Triggers Inflammatory Reaction of Endothelial Cells. Nature.

[8] Geiser T (1993). The Interleukin-8-Related Chemotactic Cytokines GRO Alpha, GRO Beta, and GRO Gamma Activate Human Neutrophil and Basophil Leukocytes. Journal of Biological Chemistry.

[9] Chakraborty H, Gu H (2009). A Mixed Model Approach for Intent-to-Treat Analysis in Longitudinal Clinical Trials with Missing Values.

[10] Ginde AA, Liu MC, Camargo CA (2009). Demographic Differences and Trends of Vitamin D Insufficiency in the US Population, 1988-2004. Archives of Internal Medicine.

[11] Judd S, Tangpricha V (2008). Vitamin D Deficiency and Risk for Cardiovascular Disease. Circulation.

[12] Hollis BW (2005). Circulating 25-Hydroxyvitamin D Levels Indicative of Vitamin D Sufficiency: Implications for Establishing a New Effective Dietary Intake Recommendation for Vitamin D. Journal of Nutrition.

[13] IOM (Institute of Medicine) (2011). Dietary Reference Intakes for Calcium and Vitamin D.

[14] Priemel M, von Domarus C, Klatte TO, Kessler S, Schlie J, Meier S, Proksch N, Pastor F, Netter C, Streichert T, Püschel K, Amling M (2010). Bone Mineralization Defects and Vitamin D Deficiency: Histomorphometric Analysis of Iliac Crest Bone Biopsies and Circulating 25-Hydroxyvitamin D in 675 Patients. JBMR.

[15] Holick MF (2004). Vitamin D Importance in the Prevention of Cancers, Type 1 Diabetes, Heart Disease, and Osteoporosis. The American Journal of Clinical Nutrition.

[16] Thomas MK, Lloyd-Jones DM, Thadhani RI, Shaw AC, Deraska DJ, Kitch BT, Vamvakas EC, Dick IM, Prince RL, Finkelstein JS (1998). Relation between Serum 25-Hydroxyvitamin D Concentrations and Mean (±SE) Serum Concentrations of Parathyroid Hormone in the Study Patients. Figure 2 in Hypovitaminosis D in Medical Inpatients by Thomas MK *et al*. The New England Journal of Medicine.

[17] Hunt SC, Williams RR, Kuida H (1991). Different Plasma Ionized Calcium Correlations with Blood Pressure in High and Low Renin Normotensive Adults in Utah. American Journal of Hypertension.

[18] Jiang BB, Morimoto S, Yang J, Niinoabu T, Fukuo K, Ogihara T (1998). Expression of Parathyroid Hormone/Parathyroid Hormone-Related Protein Receptor in Vascular Endothelial Cells. Journal of Cardiovascular Pharmacology.

[19] Osto E, Fallo F, Pelizzo MR, Maddalozzo A, Sorgato N, Corbetti F, Montisci R, Famoso G, Bellu R, Lüscher TF, Iliceto S, Tona F (2012). Coronary Microvascular Dysfunction Induced by Primary Hyperparathyroidism Is Restored after Parathyroidectomy. Circulation.

[20] Kosch M, Hausberg M, Vormbrock K, Kisters K, Gabriels G, Rahn KH, Barenbrock M (2000). Impaired Flow-Mediated Vasodilation of the Brachial Artery in Patients with Primary Hyperparathyroidism Improves after Parathyroidectomy. Cardiovascular Research.

[21] Dusso AS, Brown AJ (1998). Mechanism of Vitamin D Actions and Its Regulation. American Journal of Kidney Diseases.

[22] Carlberg C, Quack M, Herdick M, Bury Y, Polly P, Toell A (2001). Central Role of VDR Conformations for Understanding Selective Actions of Vitamin D(3) Analogues. Steroids.

[23] Ebert R, Schutze N, Adamski J, Jakob F (2006). Vitamin D Signaling Is Modulated on Multiple Levels in Health and Disease. Molecular and Cellular Endocrinology.

[24] Wu-Wong JR, Nakane M, Ma J, Ruan X, Kroeger PE (2006). Effects of Vitamin D analogs on Gene Expression Profiling in Human Coronary Artery Smooth Muscle Cells. Atherosclerosis.

[25] Jones G (1998). Current Understanding of the Molecular Actions of Vitamin D. Physiological Reviews.

[26] Seibert E, Levin NW, Kuhlmann MK (2005). Immunomodulating Effects of Vitamin D Analogs in Hemodialysis Patients. Hemodialysis International.

[27] Luscher TF, Barton M (2000). Endothelins and Endothelin Receptor Antagonists: Therapeutic Consideration for a Novel Class of Cardiovascular Drugs. Circulation.

[28] Al Mheid I, Patel R, Murrow J, Morris A, Rahman A, Fike L, Kavtaradze N, Uphoff I, Hooper C, Tangpricha V, Wayne Alexander R, Brigham K, Quyyumi AA (2011). Vitamin D Status Is Associated With Arterial Stiffness and Vascular Dysfunction in Healthy Humans. Journal of the American College of Cardiology.

